# Supplementary material for: The Small RNA Universe of Capitella teleta
Source: Front Mol Biosci. 2022 Feb 25;9:802814. doi: 10.3389/fmolb.2022.802814 (PMC8915122; doi:10.3389/fmolb.2022.802814)
Supplement: Supplementary file 1 [file DataSheet1.ZIP › Supplement/known/CAPTEscaffold_28_3360.pdf]

Provisional ID	:	CAPTEscaffold_28_3360
Score total	:	26143.9
Score for star read(s)	:	3.9
Score for read counts	:	26134.3
Score for mfe	:	1.1
Score for randfold	:	1.6
Score for cons. seed	:	3
Total read count	:	51273
Mature read count	:	51194
Loop read count	:	0
Star read count	:	79

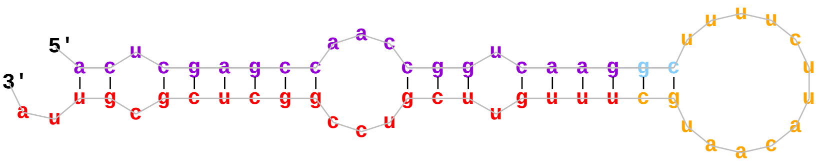

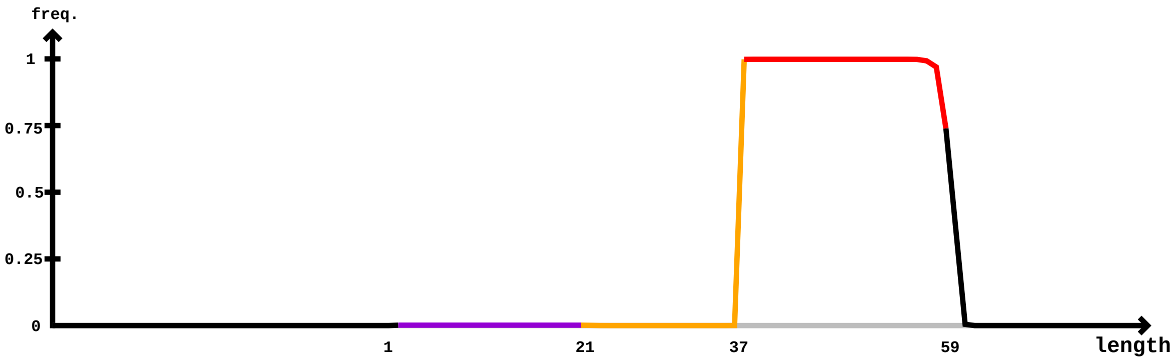


```
                                                  Star                              Mature
5'- aguuucauuuucucuucauaccacuccucaaaugacucgagccaaccggucaaggcuuuucuuacaaugcuuuguucguccggcucgcguuaugaacggaguauaugcugca  -3'   obs
    aguuucauuuucucuucauaccacuccucaaaugacucgagccaaccggucaaggcuuuucuuacaaugcuuuguucguccggcucgcguuaugaacggaguauaugcugca         exp
    aguuucauuuucucuucauaccacuccucaaaugacucgagccaaccggucaaggcuuuucuuacaaugcuuuguucguccggcucgcguuaugaacggaguauaugcugca         known
    ................((((..(((((....(((((.((((((...(((.((((((............)))))).)))...)))))).)))))....))))).)))).....  reads  mm  sample
    ..................................acucgagccaaccggucaag..........................................................  74     0   seq
    ..................................Gcucgagccaaccggucaag..........................................................  1      1   seq
    ..................................acucgagccaaccggucaaggc........................................................  2      0   seq
    ..................................acucgagccaaccggucaaggcu.......................................................  2      0   seq
    ....................................................................Ucuuuguucguccggcucgcguu.....................  1      1   seq
    .....................................................................cuuuguucguccggcucgcguu.....................  1      0   seq
    .....................................................................Guuuguucguccggcucgcguua....................  1      1   seq
    .....................................................................cuuuguucguccggcucgcguua....................  1      0   seq
    .....................................................................Auuuguucguccggcucgcguua....................  5      1   seq
    ......................................................................uuuguucguccggcucgc........................  23     0   seq
    ......................................................................uuuguucguccggcucgcg.......................  276    0   seq
    ......................................................................uuuguucguccggcuUgcg.......................  1      1   seq
    ......................................................................uuugGucguccggcucgcg.......................  1      1   seq
    ......................................................................uuuguucguccggcucgGgu......................  1      1   seq
    ......................................................................uuuguucgCccggcucgcgu......................  1      1   seq
    ......................................................................uuuguuAguccggcucgcgu......................  3      1   seq
    ......................................................................uuuguucCuccggcucgcgu......................  1      1   seq
    ......................................................................uuuguucguccUgcucgcgu......................  2      1   seq
    ......................................................................Guuguucguccggcucgcgu......................  1      1   seq
    ......................................................................uuuguucgucUggcucgcgu......................  1      1   seq
    ......................................................................uuuguucguccggcucgcgA......................  24     1   seq
    ......................................................................Auuguucguccggcucgcgu......................  3      1   seq
    ......................................................................uuuguucguccggcucgcgG......................  21     1   seq
    ......................................................................uuugAucguccggcucgcgu......................  1      1   seq
    ......................................................................uuuguucguccggcucgcgu......................  1132   0   seq
    ......................................................................uuuguucgCccggcucgcguu.....................  2      1   seq
    ......................................................................uuuguucguccgUcucgcguu.....................  3      1   seq
    ......................................................................uuuguucguAcggcucgcguu.....................  1      1   seq
    ......................................................................uuuguucguccggcuUgcguu.....................  4      1   seq
    ......................................................................uuuguucguccggcuAgcguu.....................  2      1   seq
    ......................................................................uuuguucguccggcCcgcguu.....................  1      1   seq
    ......................................................................uuugAucguccggcucgcguu.....................  5      1   seq
```

```
                                  Star                                  Mature
aguuucauuuucucuucauaccacuccucaaaugacucgagccaaccggucaaggcuuuucuuacaaugcuuuguucguccggcucgcguuaugaacggaguauaugcugca
......................................................................uuuguucguccgAcucgcguu.....................    1      1    seq
......................................................................uuuguucguccggcucgcAuu.....................    3      1    seq
......................................................................uuuguucguccggcucgcgAu.....................    1      1    seq
......................................................................uuAguucguccggcucgcguu.....................    4      1    seq
......................................................................uuuguucguccggcucgcguA.....................    6      1    seq
......................................................................uuuguucgAccggcucgcguu.....................    3      1    seq
......................................................................uuuguucguccggcuGgcguu.....................    1      1    seq
......................................................................uuuguucguccggcucgAguu.....................    6      1    seq
......................................................................Guuguucguccggcucgcguu.....................    5      1    seq
......................................................................Cuuguucguccggcucgcguu.....................    1      1    seq
......................................................................uuuAuucguccggcucgcguu.....................    1      1    seq
......................................................................uGuguucguccggcucgcguu.....................    1      1    seq
......................................................................uuuguucguccggcucAcguu.....................    3      1    seq
......................................................................uuuguucguccggcucgcgCu.....................    4      1    seq
......................................................................uuuguucguccggAucgcguu.....................    1      1    seq
......................................................................uAuguucguccggcucgcguu.....................    3      1    seq
......................................................................uuuguuUguccggcucgcguu.....................    5      1    seq
......................................................................uuuguucgGccggcucgcguu.....................    1      1    seq
......................................................................uuuguucguccggcucgcguG.....................    12     1    seq
......................................................................uuuguucguccggcucgcguN.....................    1      1    seq
......................................................................uuuguucguccggcucgcguC.....................    1      1    seq
......................................................................Auuguucguccggcucgcguu.....................    27     1    seq
......................................................................uuuguucAuccggcucgcguu.....................    4      1    seq
......................................................................uuugGucguccggcucgcguu.....................    1      1    seq
......................................................................uuuguucguccAgcucgcguu.....................    6      1    seq
......................................................................uuuguCcguccggcucgcguu.....................    1      1    seq
......................................................................uuuguucgucUggcucgcguu.....................    8      1    seq
......................................................................uuuguucguccggcucgcguu.....................    11633  0    seq
......................................................................uuuguucgucAggcucgcguu.....................    2      1    seq
......................................................................uuuguAcguccggcucgcguu.....................    5      1    seq
......................................................................uuugCucguccggcucgcguu.....................    2      1    seq
......................................................................uuCguucguccggcucgcguu.....................    2      1    seq
......................................................................uuuUuucguccggcucgcguu.....................    1      1    seq
......................................................................uCuguucguccggcucgcguu.....................    1      1    seq
......................................................................uuuguucguccggGucgcguu.....................    2      1    seq
......................................................................uuuguucguccggUucgcguu.....................    2      1    seq
......................................................................uuuguucguUcggcucgcguu.....................    1      1    seq
......................................................................uuuguuAguccggcucgcguu.....................    22     1    seq
......................................................................Nuuguucguccggcucgcguu.....................    1      1    seq
......................................................................uuuguucguccggcGcgcguua....................    4      1    seq
......................................................................uuuguucgAccggcucgcguua....................    10     1    seq
......................................................................uuuguucguccggcucCcguua....................    2      1    seq
......................................................................uuuguucguccggcucgcguCa....................    39     1    seq
......................................................................uuAguucguccggcucgcguua....................    8      1    seq
......................................................................uuuguucguccgAcucgcguua....................    5      1    seq
......................................................................uuuguucCuccggcucgcguua....................    3      1    seq
......................................................................uuuguucguccggcucgcgAua....................    4      1    seq
......................................................................uuuguucguccggcucgAguua....................    6      1    seq
......................................................................uuuguucguccggcucgcguAa....................    5      1    seq
......................................................................Auuguucguccggcucgcguua....................    75     1    seq
......................................................................uuuguucgucAggcucgcguua....................    6      1    seq
......................................................................uuuguucguccggcucgcguGa....................    2      1    seq
......................................................................uuugAucguccggcucgcguua....................    21     1    seq
......................................................................uuuguucguccggcuAgcguua....................    6      1    seq
......................................................................uuuguucguccggcucgcguuG....................    13     1    seq
......................................................................uuuAuucguccggcucgcguua....................    5      1    seq
......................................................................uuuguucguccUgcucgcguua....................    4      1    seq
......................................................................uuuguucguccggcucUcguua....................    1      1    seq
......................................................................Cuuguucguccggcucgcguua....................    11     1    seq
......................................................................uuuguuUguccggcucgcguua....................    6      1    seq
......................................................................uuuguucgGccggcucgcguua....................    3      1    seq
......................................................................uuuguucguccggcucgcguuU....................    309    1    seq
......................................................................uuuguucguccggcucgcUuua....................    1      1    seq
......................................................................uAuguucguccggcucgcguua....................    10     1    seq
......................................................................uuuguCcguccggcucgcguua....................    5      1    seq
......................................................................uuuUuucguccggcucgcguua....................    2      1    seq
......................................................................uuuguucguccgUcucgcguua....................    1      1    seq
......................................................................uuuguGcguccggcucgcguua....................    2      1    seq
......................................................................uuuguucguccggcAcgcguua....................    7      1    seq
......................................................................uuuguucguccggcucgcgCua....................    1      1    seq
```

```
                                  Star                                   Mature
aguuucauuuucucuucauaccacuccucaaaugacucgagccaaccggucaaggcuuuucuuacaaaugcuuuguucguccggcucgcguuaugaacggaguauaugcugca
.......................................................................uuuguucguccggGucgcguua....................  2      1  seq
.......................................................................Guuguucguccggcucgcguua....................  34     1  seq
.......................................................................uuuguucguccggcucAcguua....................  7      1  seq
.......................................................................uuuguucguAcggcucgcguua....................  8      1  seq
.......................................................................uuuguucAuccggcucgcguua....................  11     1  seq
.......................................................................uuugGucguccggcucgcguua....................  3      1  seq
.......................................................................uuuguAcguccggcucgcguua....................  9      1  seq
.......................................................................uuuguucguccgCcucgcguua....................  1      1  seq
.......................................................................uuuguucguccggcucgUguua....................  4      1  seq
.......................................................................uuuguucgucGggcucgcguua....................  1      1  seq
.......................................................................uuuguucguccggcucgcAuua....................  4      1  seq
.......................................................................Nuuguucguccggcucgcguua....................  16     1  seq
.......................................................................uuuguucUuccggcucgcguua....................  2      1  seq
.......................................................................uuuguucguccggcucgGguua....................  1      1  seq
.......................................................................uuuguucguccggcCcgcguua....................  2      1  seq
.......................................................................uuCguucguccggcucgcguua....................  4      1  seq
.......................................................................uuuguucguGcggcucgcguua....................  1      1  seq
.......................................................................uCuguucguccggcucgcguua....................  2      1  seq
.......................................................................uuuguucguccAgcucgcguua....................  23     1  seq
.......................................................................uuuguucgucUggcucgcguua....................  33     1  seq
.......................................................................uuuguuAguccggcucgcguua....................  81     1  seq
.......................................................................uuuguNcguccggcucgcguua....................  2      1  seq
.......................................................................uuuguucguUcggcucgcguua....................  4      1  seq
.......................................................................uuuguucguccggcucgcgGua....................  4      1  seq
.......................................................................uuuguuGguccggcucgcguua....................  2      1  seq
.......................................................................uuuguucguccggcucgcguuN....................  1      1  seq
.......................................................................uuuguucguccggAucgcguua....................  5      1  seq
.......................................................................uuuguucguccggcuUgcguua....................  26     1  seq
.......................................................................uuugCucguccggcucgcguua....................  3      1  seq
.......................................................................uuuguucgCccggcucgcguua....................  7      1  seq
.......................................................................uuuguucguccggcucgcguua....................  35029  0  seq
.......................................................................Guuguucguccggcucgcguuau...................  1      1  seq
.......................................................................uuuguucguccAgcucgcguuau...................  1      1  seq
.......................................................................uAuguucguccggcucgcguuau...................  1      1  seq
.......................................................................uuugAucguccggcucgcguuau...................  2      1  seq
.......................................................................uuuguAcguccggcucgcguuau...................  1      1  seq
.......................................................................uuuguucguccggcuUgcguuau...................  6      1  seq
.......................................................................uuuguucgAccggcucgcguuau...................  1      1  seq
.......................................................................uuuguucguccggcuGgcguuau...................  1      1  seq
.......................................................................Cuuguucguccggcucgcguuau...................  23     1  seq
.......................................................................uuuguuUguccggcucgcguuau...................  1      1  seq
.......................................................................uuuguucguccggcucgcguuaC...................  7      1  seq
.......................................................................uuuguucguccggcucgcguuau...................  1670   0  seq
.......................................................................uuAguucguccggcucgcguuau...................  1      1  seq
.......................................................................uuuguucgucUggcucgcguuau...................  1      1  seq
.......................................................................uuuguucguccggcucgcguuGu...................  1      1  seq
.......................................................................Auuguucguccggcucgcguuau...................  4      1  seq
.......................................................................uuuguucguccggcucgcguuUu...................  1      1  seq
.......................................................................uuuguucguccggcucgcguuauU..................  236    1  seq
.......................................................................uuuguucguccggcucgcguuauA..................  3      1  seq
.......................................................................uuuguucguccggcucgcguuauC..................  4      1  seq
........................................................................uguucguccggcucgcguu......................  3      0  seq
........................................................................uguucguccggcucgcguua.....................  12     0  seq
```
